# Dominant Yeast Community in Raw Sheep’s Milk and Potential Transfers of Yeast Species in Relation to Farming Practices

**DOI:** 10.3390/ani10050906

**Published:** 2020-05-22

**Authors:** Álvaro Rafael Quintana, José Manuel Perea, Beatriz García-Béjar, Lorena Jiménez, Ana Garzón, Ramón Arias

**Affiliations:** 1 Instituto Regional de Investigación y Desarrollo Agroalimentario y Forestal de Castilla La Mancha (IRIAF), CERSYRA de Valdepeñas, 13300 Ciudad Real, Spain; ljimenez@quesomanchego.es (L.J.); rarias@jccm.es (R.A.); 2 Departamento de Producción Animal, Universidad de Córdoba, 14071 Córdoba, Spain; pa2pemuj@uco.es (J.M.P.); pa1gasia@uco.es (A.G.); 3 Departamento de Química Analítica y Tecnología de los Alimentos, Facultad de Ciencias y Tecnologías Químicas, Universidad de Castilla-La Mancha, 13071 Ciudad Real, Spain; Beatriz.GBermejo@uclm.es

**Keywords:** fungi, dairy farm environment, ewe’s milk, farming practices, Manchega breed

## Abstract

**Simple Summary:**

Achieving high-quality milk is a major objective of the dairy sector in Spain, and is particularly important for the agricultural economy of the country. The dairy farm environment can affect the microbial communities in milk, and for this reason the yeast community present in this environment could have an important role in the quality of the milk and in dairy products. The focus of this study was Castilla-La Mancha, Spain. The results showed that milk contamination from the yeast species present in the dairy farm environment is reasonable, and certain farming practices favour the presence of desirable microbiota in milk.

**Abstract:**

Yeasts are always present in any type of cheese, as well as in the factories where it is produced. However, the role of the yeast community in the cheese making process, as well as the routes of contamination used by yeast species to contaminate milk from the dairy farm environment, are not well known. The objectives of this study were to broaden the knowledge of the dominant yeast community in Manchega sheep’s milk and to assess the contamination routes of the yeast species depending on the farm practices. Milk, teat surface (collected from ten ewes per farm), feed, and air (collected in milking parlours and livestock housing) samples were collected from 12 typical farms in Castilla-La Mancha, Spain with differences in farming practices, and the yeast species were identified using DNA sequencing methods. To evaluate whether certain farming practices have an effect on the distribution of species of yeast in the milk samples, a mixed model was used. The results showed that most of the dominant yeast species (mainly belonging to the genus *Candida*) found in milk were also found in the other samples, indicating a microbial transfer from the farm environment to the milk. Furthermore, the statistical model showed that factors influencing yeast counts in milk were the presence of yeasts in the milking parlour, the use of silage, and the frequency of acid treatment for cleaning the milking machines. In conclusion, milk contamination from the yeast species present in the dairy farm environment is related to certain farming practices such as the use of silage and the daily use of acid in the cleaning of the milking machines, which favours the presence of desirable microbiota in milk.

## 1. Introduction

The dairy sheep industry in Spain is particularly important for the agricultural economy of the country, producing around 400,000 tons of milk, which represents 5.7% of the total national milk production. Despite its low national percentage, sheep’s milk from Spain accounts for 18% of the total European production. Currently, Spain is the third largest producer of sheep’s milk in Europe [1]. Castilla-La Mancha is a Spanish region where sheep farming is traditionally pasture-based [2]. There, a mixed sheep-cereal system has been developed based on the Manchega breed, whose main objective is the production of milk for the manufacture of cheese under the Protected Designation of Origin “Manchego Cheese”. An in-depth description of the production system can be found in Rivas et al. [3]. In 2018, there were 547,737 Manchega ewes in milking, distributed among 665 farms [4].

In this context, achieving high-quality milk has become one of the main objectives of the dairy sector, in order to protect food safety, as well as providing the industry with milk in adequate conditions for processing [5]. The dairy farm environment should affect the microbial communities in the milk [6], and for this reason the microorganisms present in this environment and the factors influencing bulk tank milk quality [7] could have an important role in the quality of the milk and in milk products. However, the ability of the airborne microbial community to contaminate the milk remains an ongoing concern, and the sources of contamination for those microorganisms that might affect the natural microbiota of milk have not been studied adequately in dairy cattle [8], and have not been studied at all in small ruminants. Adequately healthy environmental conditions could reduce the level of the microbial communities on the farm. This is mainly due to the air present in the environment, which is the transport vehicle of matter particles and bioaerosols [9]. Although the air is a hostile environment as a habitat for microorganisms, it is important to pay special attention to the quality of this air as one important vehicle for dissemination [10].

The microbiological quality of milk has important repercussions on processed dairy products, in particular in the cheese making process. This microbiota is characterised by the presence of a wide variety of bacteria, yeasts and moulds, which interact to play a decisive role during its manufacture and ripening, improving the final flavour of the cheese and contributing to aroma development [11,12]. However, other undesirable microorganisms could also be present in the milk, increasing the risk for public health [13]. Fungal diversity in the dairy farm environment is relatively unknown compared to bacterial biodiversity [14]. Yeasts are present in any type of cheese as well as in cheese making factories, and while they contribute significantly to ripening, their role in elaboration is not well understood [15]. Certain studies have shown that the dominant yeast species of artisan cheeses mainly belong to the genus *Debaryomyces*, *Geotrichum*, *Kluyveromyces*, *Candida*, *Pichia* and *Yarrowia* [16,17,18,19], although the prevalence of certain species could be influenced by certain cheese varieties [20]. In another similar study of the yeast population in Torta del Casar cheese P.D.O. (Protected Denomination of Origin) [21], it was discovered that the dominant genera were *Pichia*, *Candida* and *Yarrowia*, which play an important role in cheese ripening. It has been demonstrated that the presence of yeasts could have a positive influence on the cheese properties. However, high concentrations of yeast could lead to cheese deterioration and the production of unpleasant odours and flavours [15]. For these reasons, it is important to improve our knowledge about the yeast community present in the dairy farm environment, which could affect the milk and dairy products.

Therefore, the aims of the present work were to study the dominant yeast communities in Manchega ewes’ milk and to explore the contamination routes used by those microorganisms in relation to farm facilities and farming practices. The data compiled under this study provide reliable, essential information regarding the distribution and transmission of the dominant species of yeasts on sheep dairy farms. They should, therefore, serve as the basis for decisions regarding the prevention and control of these populations in relation to animal health and milk quality.

## 2. Materials and Methods 

### 2.1. Population and Study Design

Twelve typical farms with dairy systems representative of the main milking and feeding practices in Castilla-La Mancha were selected from among those belonging to the National Association of Selected Sheep Breeders of the Manchega Breed (AGRAMA). Due to the fact that environmental microorganisms are very labile, the farms had to be less than two hours away from the Dairy Laboratory of CERSYRA in Valdepeñas (Ciudad Real, Spain) for the sampling analysis.

Farmers were asked questions about their milking practices, estimated number of milking ewes, milk production, sanitary conditions of the flock, general management, and animal feeding. In addition, the orientation and size of the milking parlour and the livestock housing, the number of openings in these buildings, the handling and characteristics of the milking machines, and the environmental conditions during milking were referenced during sampling. The differences among farms were mainly in the dairy infrastructure, milking and cleaning protocols, as well as silage use and use of grain during milking.

The farms were visited once every three months during 2018 to take samples of milk, teat surface, feed and air from the livestock housing and the milking parlour. In each sampling, an air sample was collected during milking in the milking parlour. Another air sample was collected in the livestock housing, where the milking ewes were resting before milking. Air samples were collected using the AirPort MD8 air sampler (Sartorius Stedim Biotech, Sartorius, Goettingen, Germany), which operates according to the filtration and impact methods. A defined air volume, 1000 L, was passed through a gelatin membrane filter (Sartorius Stedim Biotech, Sartorius, Goettingen, Germany) where yeasts are retained. Then, the filters were placed directly on a suitable culture medium in a Petri dish to carry out the counts. The air sampler was placed at a distance of at least 1 m above the floor and 1 m away from walls or obstacles [22], and in the case of the milking parlour, the sampler was also placed near the clusters.

During milking, ten ewes were randomly selected and the surface of both their teat was sampled before coming into contact with the cluster, according to the research carried out by Vacheyrou et al. [8]. A sterile wipe was used per ewe, moistened with sterile saline solution (0.9%) and collected in a sterile plastic bag.

Milk was sampled in the bulk tank after milking in two sterile 50 mL containers after at least 5 min of milk homogenization, and transferred into a sterile plastic box stored in a polystyrene refrigerated box for conservation. Lastly, around 150 g of the animal feed was also collected in a sterile plastic bag during each visit to the farm.

A total of 240 samples (48 milking parlour air samples, 48 livestock housing air samples, 48 bulk tank milk samples, 48 teat surface samples and 48 animal feed samples) were taken in order to analyse the yeast community on the farms.

### 2.2. Sample Handling and Microbial Count

For all the samples analysed, Yeast Extract Peptone Dextrose (YPD) plates (Pronadisa) were selected for yeast counts which were incubated at 25 °C for 48 h. Following Pérez-Martín et al. [23], some biphenyl crystals (approximately 100 mg per plate) (Sigma) were added to the cover plates to avoid the development of moulds. In the case of air samples, the gelatin membrane filters were directly placed in duplicate on YPD plates and incubated. 

The sterile wipes used for the teat samples were added to 50 mL of sterile saline solution (0.9%) and were mixed vigorously in a Stomacher (IUL S.A.) for one minute. After that, serial dilutions were carried out and samples were inoculated on agar plates in duplicate. The animal feed samples (10 g) were homogenised with 90 mL of sterile saline solution (0.9%) in a Stomacher, and milk samples did not require any special handling. For sampling inoculation, the same procedure as described for teat samples was carried out.

Milk samples were stored at 4 °C during the 2 h after sampling until analysis, and did not require any special handling. Dilutions were done and 100 microlitres of the liquid was inoculated on the agar medium.

After incubation, colonies were counted and the results were expressed as colony forming units (CFU), given by the average of the numbers obtained in the two repeat samples. Depending on the samples, the results were expressed in different ways following the indications of Wehr and Frank [24]: in log_10_ CFU per mL of milk, in log_10_ CFU per g of animal feed, in log_10_ CFU per wipe for teat surface and in log_10_ CFU per 1000 L of air for the air samples.

### 2.3. Isolation of Yeasts and Typing of Yeast Isolates by RAPD-PCR

A representative number (10%) of colonies grown on all the sample plates were randomly picked and propagated until purification on YPD plates. Pure cultures were stored at −80 °C in YPD broth containing 20% (*v*/*v*) glycerol (Panreac, Barcelona, Spain).

The cells were harvested by centrifugation (5000 rpm for 5 min) and were washed with sterile 0.9% saline solution. Recovered pellet was immediately treated with a zymolyase solution (10 mg/mL zymolyase 20 T in 1.2 M sorbitol buffer, 40 mM sodium phosphate buffer, pH = 7), as described by Fernandez-Pacheco et al. [25]. After incubation at 37 °C/30 min and 95 °C/5 min, a cell lysate was obtained. RAPD-PCR reaction was accomplished using the M13 primer (5’-GAGGGTGGCGGTTCT-3’) performed as described in Fadda et al. [26] with minor modifications using a PCR (Thermal Cycler, Applied Biosystems, Waltham, MA, USA). The final volume reaction, 20 µL, contained 0.3 µL rTaq (5U) DNA polymerase, 0.4 µL dNTP mix (0.2 mM) (Bioline), 2 µL buffer (Bioline), 1.2 µL MgCl_2_ (3 mM) (Bioline), 2.5 µL M13 primer (Metabion International, Munich, German) and 1 µL of genomic DNA. PCR amplification conditions were as follows: 2 cycles at 94 °C for 1 min, 45 °C for 1 min and 72 °C for 1 min, followed by 35 cycles at 94 °C for 40 s, 52 °C for 1 min and 72 °C for 3 min with a final extension step at 72 °C for 10 min. The PCR products were resolved by electrophoresis on 2% agarose gel for 3 h, stained with RedSafe (INtRON Biotech, Seoul, Korea) and visualised using a gel documentation system (Gel Doc^TM^ XR 170-8171, BioRad, Marnes-la-Coquette, France), where DNA fragment sizes were determined in comparison to the 100-bp DNA ladder (Biotools, Madrid, Spain).

The similarity of patterns was expressed by the Pearson correlation coefficient (r), and clustering was performed by the unweighted pair group method using average linkage (UPGMA; Gel Compar, Comparative Analysis of Electrophoresis Patterns, Version 4.0, Applied Maths, Kortrijk, Belgium) as Vauterin and Vauterin [27] described.

A reproducibility study to determine the minimum percentage of similarity necessary for strain discrimination was carried out on six isolates and with four iterations of the entire procedure beginning with culture inoculation. Each isolate was grown in four separate cultures from which DNAs were extracted and amplified. The amplification products obtained for two replicates of each isolate were run on one gel, and those for the other two replicates were run on another different gel to estimate gel effects. The level of similarity (r) obtained between repeats, when included within the dendrogram for all strains, established a discrimination threshold below which patterns were considered to be different.

### 2.4. Identification of Isolates

Based on pattern similarity, 10% of the isolates included in each RAPD group as well as those which had a unique pattern were analysed. A total of 88 representative isolates from the 633 total yeasts were identified by MALDI-TOF using a VITEK MS instrument (bioMérieux, Marcy l’Etoile, France) in the facilities of Probisearch (Tres Cantos, Madrid, Spain). Identification was defined as a 99%–100% match to the species-specific *m/z* profiles in the database.

### 2.5. Biodiversity Study of Yeasts

Simpson’s Diversity Index was calculated in order ascertain the richness of species on each farm. This coefficient is calculated based on the number of species present and the relative abundance of each among the total yeasts found. As richness of species and evenness increase, so diversity increases. The following equation was used:

(1) $$D=1-\left(\frac{\sum n\left(n-1\right)}{N\left(N-1\right)}\right)$$

where D is the Simpson’s Diversity Index; n is the total number of organisms of a species; and N is the total number of organisms of all species in the same environment. The calculated value of D ranges between 0 (no diversity) and 1 (infinite diversity).

Moreover, the biodiversity percentage was calculated with the aim of evaluating the genetic diversity in the species found by comparing the number of strains identified with the number of yeast isolates per species, where higher values indicate higher strain biodiversity.

### 2.6. Statistical Analysis

Prior to carrying out statistical analyses, yeast counts (YM) were normalised by log_10_-transformation. A mixed model (MIXED procedure in SAS version 9.1, SAS Institute Inc., Cary, NC, USA) was used to examine the factors that influence log YM variation [7]. The model could be expressed as:

(2) $$\mathrm{YM}=\overset{n}{\underset{i=1}{\sum }}\beta_{i}f_{i}+\delta$$

where YM is the log_10_ of yeast count in milk, *β_i_* are the unknown parameters to be estimated, *f_i_* are the explanatory variables and *δ* is the error term.

The factors evaluated were the following: presence of yeasts in the air from the milking parlour (YA1: yes or no), presence of yeasts in the air from the livestock housing (YA2: yes or no), presence of yeast on the surface of teat (YT: yes or no), presence of yeast in the feed (YF: yes or no), season (spring, summer, autumn or winter), hygiene of the milking parlour (HygMP: adequate or not), hygiene of the livestock housing (HygLH: adequate or not), orientation of the milking parlour (OriMP: north-south, east-west, northeast-southwest, northwest-southeast, among others) orientation of the livestock housing (OriLH: north-south, east-west, northeast-southwest, northwest-southeast, among others), ventilation of the livestock housing (VentLH: adequate or not), milkline of the milking machine (Milkline: low-level or mid-level system), frequency of cleaning of the milking parlour (CleanMP: daily, twice per day or more), frequency of changing of milk filters (Filter: after each milking, daily or every two days), possibility of contact between the clusters and the ground (Cluster: yes or no), frequency of use of acid for cleaning the milking machine (Acid: daily, each 2–3 days, or less frequently), use of silage (Silage: yes or no) and use of grain during milking (Grain: yes or no). The conditions used to determine the inadequate hygiene of the milking parlour were the presence of airborne dust particles, the presence of litter on the floor and the accumulation of waste; the conditions used to determine a inadequate hygiene in the livestock housing were bad litter management, presence of airborne dust particles and leftover in the feeders; and the conditions used to determine inadequate ventilation in the livestock housing were the presence of ammonia odour and the absence of sufficient windows and doors for providing a good ventilation.

To avoid problems derived from the small sample size (48 complete cases), we used the following analytical sequence [28]. Firstly, the strength of the bivariate association between factors and log YM was quantified performing ANOVA (factors with three or more levels) or Student’s t- tests (factors with two levels). Factors significantly associated with log YM (*p* < 0.20) were considered candidate variables to include in the mixed model. Secondly, a manual model-building selection was conducted for the development of the mixed model. All main effects were evaluated as potential predictors. A *p*-value of <0.05 was used as the retention criterion [29]. As a first step, we compared all the possible models with just one variable using the Akaike Information Criteria (AIC) value. To the model with the lowest AIC value and one predictor, we added all the remaining factors one by one and compared them based on the AIC value. This process was repeated until the model with the lowest AIC was obtained [30]. This was considered the most plausible one, and was selected as the final model. Once the model was defined, the degree of collinearity was analysed using the variance inflation factor (VIF) of the regression coefficients. It was considered that there was a serious multicollinearity problem if any of the VIFs was greater than 10 [31]. The Kolmogorov-Smirnov test was used to verify normality in the distribution of the residues, the Durbin-Watson test to detect the absence of autocorrelation of the residues, and heteroscedasticity was evaluated by the White test. The model fit was determined by the adjusted coefficient of determination, the mean absolute error and the mean absolute percentage error [32,33]. 

## 3. Results

### 3.1. Identification of Yeasts

A total of 633 isolates from the samples were typed. With a reproducibility between different RAPD-PCR patterns for the same isolate of >86%, 88 representative isolates from all the samples were obtained and identified by MALDI-TOF. With a probability of identification of >99%, these representative isolates belonged to 11 species of genus *Candida*, which was present in all the areas sampled. A representative isolate was identified as *Rhodotorula mucilaginosa* in only one case, having appeared on the teat surface. Several yeast species may not have been detected because of their low counts in samples and/or the PCR biases encountered in such molecular fingerprinting techniques.

The predominant species was *C. parapsilosis*, with a percentage of 57%. This species was identified, and the same genotypes were found in the different areas sampled. The species *C. famata*, *C. krusei* and *C. guilliermondii* were also important, with percentages of 13%, 12% and 6%, respectively, and the same genotypes were found in milk, animal feed and teat surface. Despite the lowest percentage of the other species, *C. kefyr* (3%) established a relationship between the milking parlour, livestock housing and animal feed, because the same genotypes were found in both air and feed. The species identified and the relationships found between the areas are presented in Table 1.

Overall, the air in the milking parlour and the livestock housing was less contaminated than in other places (Table 2). Air is an important vehicle of dissemination, but it is also a hostile environment as a habitat for microorganisms because they are stressed by the lack of nutrients and by dehydration. However, *C. kefyr* showed a relationship between the air in both buildings on the farm, and *C. inconspicua* established a relationship between the milk and the air in the milking parlour. 

Most of the yeast species in milk, such as *C. famata*, *C. parapsilosis*, *C. guilliermondii* and *C. krusei*, were also identified in the animal feed and the teat surface, suggesting a link between the feed, teat hygiene and the milk. In addition, *C. catenulata* showed that there is a connection between the milk and the teat surface, a relationship that was already shown by Vissers et al. [34], who claimed that correct management of the farm could reduce the transmission of contamination to the milk. 

Finally, the other species only appeared in one sampled area: *C. lambica*, *C. holmii* and *C. rugose* in the feed, and *C. zeylanoides* and *Rh. mucilaginosa* on the teat surface, as indicated in Table 1.

### 3.2. Biodiversity Study of Yeasts

**Figure 1:**
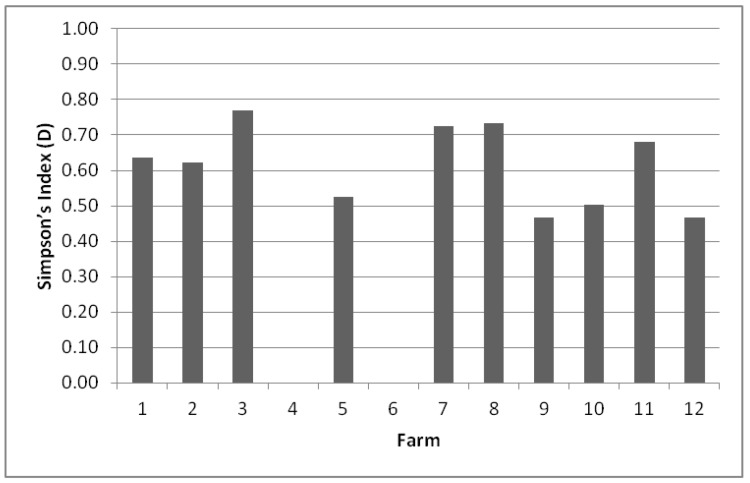
Simpson’s Index (D) biodiversity of species present on each farm. The value ranges between 0 and 1, where 1 represents infinite diversity and 0 no diversity.

The biodiversity on each farm was evaluated by calculating Simpson’s index (D, between 0 and 1) (Figure 1). D values in the range between 0.5 and 1 were obtained for 8 of the 12 environments studied.

The greatest richness of species was found on farm F3 (0.769), with seven species identified, followed by farms F8 (0.733) and F7 (0.725), which presented seven and four species, respectively. The other farms which showed a good D index, ranging between 0.500 and 0.700, were F11 (0.682), F1 (0.636) and F2 (0.621), but they presented a smaller number of identified species. However, it was observed that two farms had non-existent diversity owing to the fact that there was a predominant species, i.e., on farms F6 and F4, only *Candida parapsilosis* was isolated from the samples. 

**Figure 2:**
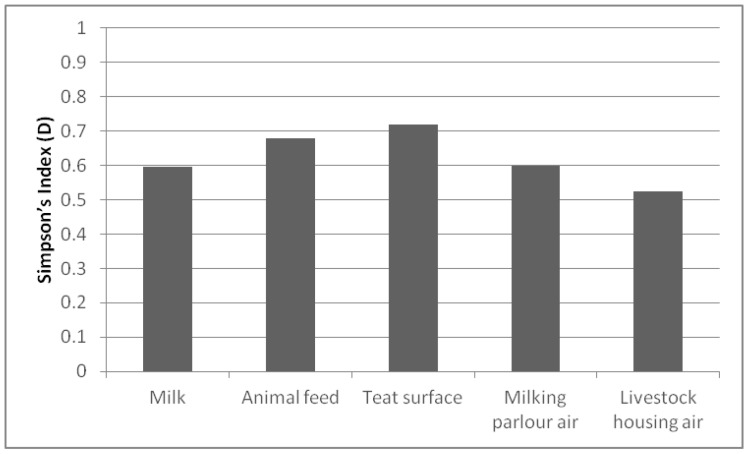
Simpson’s Index (D) biodiversity of species present in each sample.

Regarding the richness of species per sample (Figure 2), the D index was higher than 0.500 in all of them. The sample that presented the highest value was the teat surface (0.719), followed by the animal feed (0.679). In contrast, the air in the livestock housing showed the lowest D index (0.524). Since transmission of species is easier between samples from the same environment, better biodiversity results were found in the studied samples than on the farms.

**Figure 3:**
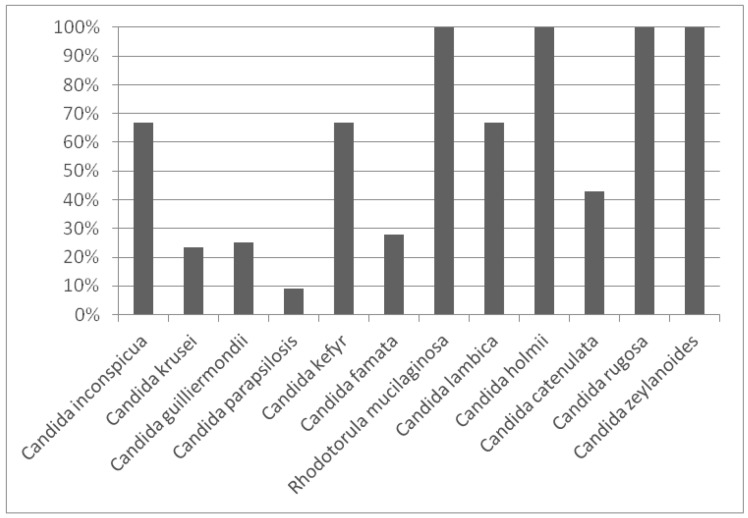
Genetic diversity of each species isolated from Manchega dairy farms environments.

Additionally, an analysis of genetic diversity was carried out by computing the biodiversity percentage of each species. The results are outlined in Figure 3, with values ranging between 0% and 100%.

The highest biodiversity was identified in four species (*C. holmii*, *C. rugosa*, *C. zeylanoides* and *Rh. mucilaginosa*). In contrast, the species which presented the greatest number of isolates (*C. parapsilosis*) also showed the smallest genetic diversity (9%) due to the fact that one strain was predominant in most of the samples analysed. In general, those species with five or more isolates exhibited a biodiversity percentage lower than 50%. 

### 3.3. Factors Related to The Concentration of Yeasts in Milk

The main characteristics of the farms as collected from the farmers’ responses to the questionnaire are presented in Table 3.

**Figure 4:**
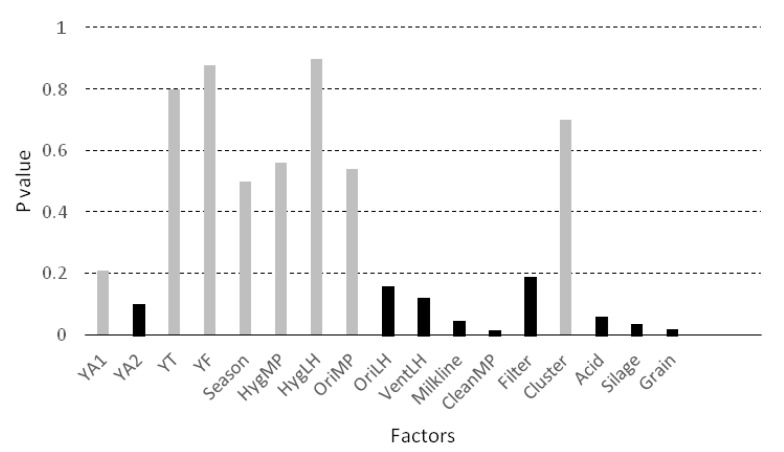
Bivariate association (ANOVA or Student’s t-test) between the concentration of yeasts in the milk (log_10_ CFU/mL) and the considered factors. Legend: YA1 = presence of yeasts in the air from the milking parlour, YA2 = presence of yeasts in the air from the livestock housing, YT = presence of yeast on the surface of teat, YF = presence of yeast in the feed, Season = season of the year, HygMP = hygiene of the milking parlour, HygLH = hygiene of the livestock housing, OriMP = orientation of the milking parlour, OriLH = orientation of the livestock housing, VentLH = ventilation of the livestock housing, Milkline = milkline of the milking machine, CleanMP = frequency of cleaning of the milking parlour, Filter = frequency of changing of milk filters, Cluster = possibility of contact between the clusters and the ground, Acid = frequency of use of acid for cleaning the milking machine, Silage = use of silage, Grain = use of grain during milking.

Factors included in the bivariate analysis are presented in Figure 4. Nine variables (marked in black in Figure 4) were associated with the concentration of yeasts with a P value below 0.20, and were therefore candidate variables for inclusion in the mixed model. Four of them were significantly associated with YM in the bivariate analysis (*p* < 0.05): the milkline of the milking machine (*p* = 0.045), the frequency of cleaning of the milking parlour (*p* = 0.016), the use of silage (*p* = 0.035) and the use of grain during milking (*p* = 0.018). The use of grain, the use of silage, the low milkline configuration and more frequent cleaning of the milking parlour tend to increase the concentration of yeasts in the milk.

Table 4 shows the results obtained by the best fitting model for YM in milk from Manchega sheep dairy farms. The adjusted determination coefficient and the average absolute percentage error reached values of 40.0% and 0.94, respectively. In addition, the variance inflation factor (VIF) of the regression coefficients fluctuated between 1.03 and 1.33, so there is not a multicollinearity problem.

**Figure 5:**
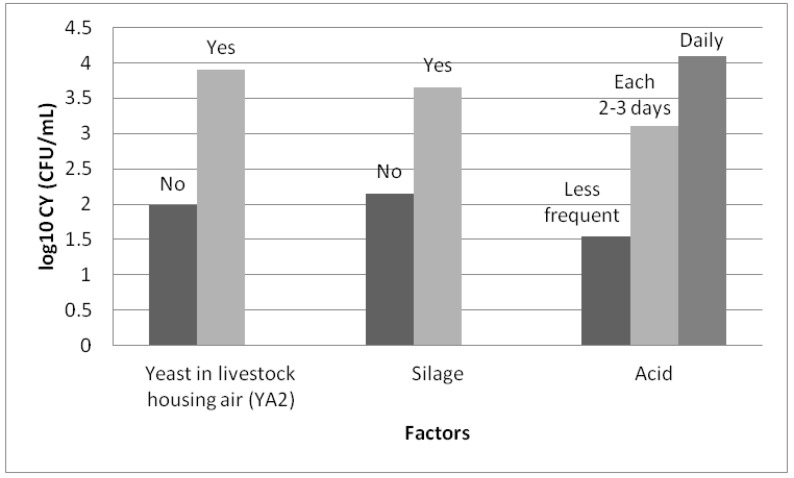
Least square means of the concentration of yeast in milk (log_10_ CFU/mL) from Manchega sheep dairy farms for factors included in the best fitting mixed model. Means within factors with different colours vary (Student–Newman–Keuls, SNK *p* < 0.05).

Three factors were included in the model: the presence of yeasts in the air in the livestock housing (*p* = 0.005), the frequency of use of acid for cleaning the milking machine (*p* = 0.005) and the use of silage (*p* < 0.001). The concentration of yeasts in the milk increases significantly with the frequency of use of acid for cleaning the milking machine, the use of silage and the presence of yeasts in the air in the livestock housing (Figure 5).

## 4. Discussion

Characterisation and dispersion of yeast communities from sheep farms were carried out in this study using innovative technologies such as MALDI-TOF and by applying statistical models and analysing biodiversity parameters. Yeast presence in farm environments has not been studied as thoroughly as bacterial communities have, especially on sheep farms. This study not only provides new information about yeast contamination on sheep farms, but also establishes a relationship between the different samples and the farms analysed. 

The achieved results of yeasts in milk, mean counts of 2155.9 CFU/mL, were in accordance with other previous research [15,35]. The MALDI-TOF technique allowed the determination of a total of 12 species, which mainly belonged to the genus *Candida*. This genus is one of the most resistant in all environments and is normally detected in farm environments, as it can be associated either with the livestock animal microbiome (mucosal tissues, gastrointestinal tract, etc.) or the dairy products [14,36]. The genus *Candida* is present in a wide variety of artisan cheeses [37], being dominant in some, such as Torta del Casar [21]. In this research, *C. parapsilosis* was not only the main species isolated, but it was also associated with all kinds of samples. *C. parapsilosis* has previously been related with dairy products as well as farm animals, so its presence in the environments studied was not surprising [14,38]. With regard to the other *Candida* species identified, these yeasts (usually opportunistic pathogens) are commonly found in raw milk from different animals and can be highly prevalent if the farm animal is suffering from mastitis [39,40]. They can even cause cases of mastitis in sheep [41], with *Candida* being the main genus involved in fungal mastitis in ruminants [42]. Nevertheless, these species can also carry out fermentation of dairy products or can be related with the ripening process of cheeses [39,43]. *Rh. mucilaginosa* was the only species that did not belong to the genus *Candida*, and other studies have pointed out its relationship with ewes’ milk [14].

The Simpson’s Index allowed the evaluation of the richness of species on each farm and in each sample studied. The results indicate that a loss of diversity is found when a predominant species exists. This could be due to the fact that some environments can be unfavourable for specific types of yeasts, but also that climatic conditions on the sampling may affect the richness of species [44]. In contrast, samples from different farms turned out to be rich environments not only because of the high nutrient content in certain samples (milk, feed, etc.), but also because other samples are a natural vehicle for yeast dispersion (air, animals, etc.). Despite the fact that *C. parapsilosis* was the most isolated species, its genetic variability proved to be poor due to the presence of a predominant genotype which seems to present the best dispersion and resistance capability. The same behaviour was noticed in *C. krusei*, *C. guillerimondii*, *C. famata and C. catenulata,* where a prevailing strain was also identified in different examples. However, common genotypes have been isolated both in different samples as well as on different farms which proves the dispersion capability of these strains, linked to the geographical area studied. As is referenced above, these species are commonly associated with the farm animal microbiome, so their transfer from teat to milk can occur easily. Moreover, the presence of common strains on different farms can be explained due to the fact that these strains have also been identified in air, which is a vector of yeast transmission and may be involved in their dispersion [45].

The statistical model showed that the factors that influence the concentration of yeasts in milk are the presence of yeasts in the livestock housing environment, the use of silage and the frequency of acid treatment for cleaning the milking machine. These results suggest that the contamination of yeasts in milk can come mainly from the feed used on the farm, as also happens in the case of bacteria of the genus *Clostridium* [46], with the presence of yeasts in livestock housing playing a relevant role. Some authors have reported a higher number of yeasts when using silage for feeding the animals, usually in winter [47]. This could be due to bad management of the silage, starting the activity of aerobic microorganisms (usually initiated by yeasts), which could produce deterioration in the feed [48]. Similar to the connection from silage to milk, in our research different strains from different species were found in both the air in the livestock housing and in the milk (*C. parapsilosis* and *C. famata*), which reinforces the idea that there is a link between this environment and milk, promoting yeast contamination.

In the same way, the results indicated that correct cleaning of the milking machine could enhance the proliferation of yeasts in the milk. It has been documented that yeasts occur in raw milk in low numbers, probably due to competition for the growth substrates by psychrotrophic bacteria of milk [49]. Maintaining correct hygiene in milking facilities improves the elimination of undesirable bacteria [50] and preserves a wider bacterial diversity in the milk, with a more balanced profile between desirable and undesirable bacteria [51]. Our results showed that the daily use of acid in the milking machines favours the presence of yeasts in milk. The frequency of acid washing was recently studied as a factor influencing bulk tank total bacterial count variation in Spanish dairy sheep flocks [7], and probably a daily use of acid detergent might eventually result in material deterioration of liners and short and long milk tubes, thus facilitating yeast proliferation and contamination, particularly when yeasts are present in the environment. For that reason, promoting good hygiene practices on dairy farms, like surveillance programs in milking equipment, could improve the organoleptic characteristics and the quality of the final product, avoiding high concentrations of undesirable bacteria which could cause unpleasant odours and flavours which are not favourable for dairy products. In addition, knowledge regarding yeast content in the air of the milking parlour is very interesting, with a view to minimizing iatrogenic contamination of the mammary gland in the moment of antibiotic dry therapy practice.

## 5. Conclusions

In this study, the contamination routes used by the dominant yeast community to affect ewes’ milk were researched in relation to farming practices. Relationships were found between the presence of yeasts in the bulk tank and in the dairy farm environment, because common genotypes were observed in the different areas (milk, milking parlour air, livestock housing air, animal feed and teat surface), with *C. parapsilosis* being the dominant species. In addition, an association between certain farming practices and the presence of yeasts in bulk tank milk were found, increasing the number of yeasts with the presence of yeasts in the livestock housing air, with the use of silage and with the daily use of acid detergents in the milking machines. An exhaustive study of the yeast communities on farms and the farm contamination routes could be recommended, as well as the microbial transfers of yeast community from milk to cheese, in particular the role of genus *Candida* in the quality of cheeses. Increasing the samples from other sources and other production systems may be taken for further studies on farm microbial transfers. Finally, the use of air samplers in analytical surveillance programs of dairy sheep flocks from the dairy industry should be taken into account, in order to monitor the air quality of the milking parlour and livestock housing, which would be very convenient in milk analytical surveillance programs.

## Figures and Tables

**Table 1:** Presence (+) or absence (−) of the different species of genetically equal yeasts identified in each kind of sample.

Species	Milk	Milking Parlour Air	Livestock Housing Air	Animal Feed	Teat Surface
*Candida inconspicua* *Candida krusei* *Candida guilliermondii* *Candida parapsilosis* *Candida lambica* *Candida holmii* *Candida famata* *Candida catenulata* *Candida rugosa* *Candida zeylanoides* *Candida kefyr* *Rhodotorula mucilaginosa*	+ + + + − − + + − − − −	+ − − + − − − − − − + −	− − − + − − + − − − + −	− + + + + + + − + − + −	− + + + − − + + − + − +

**Table 2:** Average concentration of yeasts in the different samples analysed.

Variable	Mean	Standard Deviation	Coefficient Variation
YM	2147.86	4791.61	223.09
YA1	6.56	27.04	412.00
YA2	8.12	43.64	537.17
YF	79,716.5	104,667	131.30
YT	21.15	57.02	269.55

Legend: YM = Yeast in milk (CFU/mL), YA1 = yeasts in the air from the milking parlour (CFU/1000 L), YA2 = yeasts in the air from the livestock housing (CFU/1000 L), YF = yeast in the feed (CFU/g), YT = yeast on the surface of teat (CFU/wipe).

**Table 3:** Distribution of frequencies obtained by the farmers in the applied questionnaire.

Variable	Levels	Frequency (%)
YA1	Yes	44 (91.7)
	No	4 (8.3)
YA2	Yes	44 (91.7)
	No	4 (8.3)
YF	Yes	31 (64.6)
	No	17 (35.4)
YT	Yes	3 (6.3)
	No	45 (93.7)
Season	Spring	12 (25.0)
	Summer	12 (25.0)
	Autumn	12 (25.0)
	Winter	12 (25.0)
HygMP	Adequate	35 (72.9)
	Not	13 (27.1)
HygLH	Adequate	35 (72.9)
	Not	13 (27.1)
OriMP	N-S	24 (50.0)
	E-W	8 (16.7)
	NE-SW	8 (16.7)
	NW-SE	8 (16.7)
OriLH	N-S	12 (25.0)
	E-W	16 (33.3)
	NE-SW	8 (16.7)
	NW-SE	8 (16.7)
	Other	4 (8.3)
VentLH	Adequate	24 (50.0)
	Not	24 (50.0)
Milkline	High Low	32 (66.7) 16 (33.3)
CleanMP	After each milking	25 (50.0)
	Daily	12 (25.0)
	Less frequently	12 (25.0)
Filter	After each milking	20 (41.7)
	Daily	24 (50.0)
	Every two days	4 (8.3)
Cluster	Yes	24 (50.0)
	No	24 (50.0)
Acid	Daily	8 (16.7)
	Each 2–3 days	36 (75.0)
	Less frequently	4 (8.3)
Silage	Yes	16 (33.3)
	No	32 (66.7)
Grain	Yes	24 (50.0)
	No	24 (50.0)

Legend: YA1 = presence of yeasts in the air from the milking parlour, YA2 = presence of yeasts in the air from the livestock housing, YF = presence of yeast in the feed, YT = presence of yeast on the surface of teat, Season = season of the year, HygMP = hygiene of the milking parlour, HygLH = hygiene of the livestock housing, OriMP = orientation of the milking parlour, OriLH = orientation of the livestock housing, VentLH = ventilation of the livestock housing, Milkline = milkline of the milking machine, CleanMP = frequency of cleaning of the milking parlour, Filter = frequency of changing of milk filters, Cluster = possibility of contact between the clusters and the ground, Acid = frequency of use of acid for cleaning the milking machine, Silage = use of silage, Grain = use of grain during milking.

**Table 4:** Factors associated with the concentration of yeasts in milk (log_10_ CFU/mL) from Manchega dairy sheep farms using a mixed model.

Variable	Coefficient	SD	*f* Value	*p* Value	VIF
Yeast in livestock housing air (YA2)	-	-	8.63	0.005	1.06
No	−0.94	0.32			
Yes	0.94	0.32			
Silage	-	-	13.30	<0.001	1.33
No	−0.77	0.21			
Yes	0.77	0.21			
Acid	-	-	5.90	0.005	1.03
Daily	1.19	0.35			
Each 2–3 days	0.17	0.31			
Less frequent	−1.36	0.33			

Legend: SD = Standard Error, VIF = Variance Inflation Factor.
